# Duplication and relocation of the functional *DPY19L2 *gene within low copy repeats

**DOI:** 10.1186/1471-2164-7-45

**Published:** 2006-03-09

**Authors:** Andrew R Carson, Joseph Cheung, Stephen W Scherer

**Affiliations:** 1 Department of Genetics and Genomic Biology, Hospital for Sick Children, Toronto, Ontario, Canada; 2 Department of Medical and Molecular Genetics, University of Toronto, Toronto, Ontario, Canada

## Abstract

**Background:**

Low copy repeats (LCRs) are thought to play an important role in recent gene evolution, especially when they facilitate gene duplications. Duplicate genes are fundamental to adaptive evolution, providing substrates for the development of new or shared gene functions. Moreover, silencing of duplicate genes can have an indirect effect on adaptive evolution by causing genomic relocation of functional genes. These changes are theorized to have been a major factor in speciation.

**Results:**

Here we present a novel example showing functional gene relocation within a LCR. We characterize the genomic structure and gene content of eight related LCRs on human Chromosomes 7 and 12. Two members of a novel transmembrane gene family, *DPY19L*, were identified in these regions, along with six transcribed pseudogenes. One of these genes, *DPY19L2*, is found on Chromosome 12 and is not syntenic with its mouse orthologue. Instead, the human locus syntenic to mouse *Dpy19l2 *contains a pseudogene, *DPY19L2P1*. This indicates that the ancestral copy of this gene has been silenced, while the descendant copy has remained active. Thus, the functional copy of this gene has been relocated to a new genomic locus. We then describe the expansion and evolution of the *DPY19L *gene family from a single gene found in invertebrate animals. Ancient duplications have led to multiple homologues in different lineages, with three in fish, frogs and birds and four in mammals.

**Conclusion:**

Our results show that the *DPY19L *family has expanded throughout the vertebrate lineage and has undergone recent primate-specific evolution within LCRs.

## Background

Gene duplication is an important factor in evolution. These duplications can arise during the expansion of low copy repeats (LCRs), or segmental duplications, which make up a significant proportion of the human genome sequence [1,2]. The high sequence identity (>90% over >5 kb) shared between these DNA segments indicates that they have arisen over the past 35 million years (MY) of primate evolution and in some instances are human specific. Detailed analyses of LCRs in humans have shown that these repeats are often interspersed, rather than tandemly duplicated [1,3]. LCRs are observed both interchromosomally, with enrichment in pericentromeric and subtelomeric chromosomal regions [4], and intrachromosomally, with enrichment in euchromatic sequences.

LCRs have important consequences in vertebrate gene evolution. Traditionally, the major force in genome evolution was thought to be nucleotide substitutions. However, gene duplications have been shown to significantly contribute to evolution by acting as molecular substrates for divergence [5,6]. In fact, the estimated rate of duplicate gene fixation (1/gene/100 MY) [7-9] is similar to or greater than the estimated nucleotide substitution rate at silent sites (0.1–0.5/site/100 MY) [10]. Duplicate genes arise by several different mechanisms: unequal crossing over and retroposition, which give rise to small duplications, and chromosomal duplication and whole genome polyploidization, which involve much larger amounts of DNA [11]. Considering that the last whole genome polyploidization event in the land vertebrate lineage is estimated to have occurred more than 600 MY ago [12], duplications of this nature are not expected to play a role in primate speciation. Instead, smaller duplications, such as those creating LCRs, which maintain gene structure and organization, are predicted to have significantly affected recent primate evolution. This theory is supported by the finding that segmental duplications make up a significant proportion of primate genomes, with estimates of around 5% in the human genome [1,2,4]. Although the LCR composition of mammalian genomes has been recently examined, an in-depth investigation of the gene content in LCRs and their role in gene evolution has not been undertaken.

Studies have shown an unexpectedly large complement of functional gene duplicates in most genomes [13,14], which can be accounted for by two of the fates for duplicated genes. One of these fates, subfunctionalization, occurs when both copies of a gene degenerate in a complementary fashion, such that both copies are required to perform the ancestral functions [15,16]. The other fate is neofunctionalization, in which positive selection for beneficial mutations occurs, allowing one of the gene copies to diverge rapidly and adopt a new function [17]. Subfunctionalization is predicted to develop more frequently than neofunctionalization because it exploits common degenerative mutations rather than relying on rare beneficial mutations.

The third and most common fate for copies of a gene following duplication and fixation is for one of the copies to become nonfunctional through the accumulation of deleterious mutations. These mutations are tolerated when duplicate genes are functionally redundant, relaxing the selection on at least one of the two copies. In humans, it is estimated that duplicate genes have an average half life of 7.5 MY [8]. After a copy is silenced, a nonfunctional remnant will exist until it is deleted or mutated beyond recognition. These remnants are known as pseudogenes. Pseudogenes can also be created by partial duplications that exclude key sequences or regulatory regions of genes. Several recent studies have evaluated the pseudogene content in the human genome [18,19] and have found that their number may equal or even surpass the number of functional genes.

Although pseudogenization restores the proteome to its pre-duplication state, it may passively affect adaptive evolution. Since selection is often relaxed on both copies of the gene after duplication, either the ancestral or the progeny copy can be inactivated. Pseudogenization of the ancestral copy constitutes gene relocation. Much like larger rearrangements, relocation of unique genes to different chromosomes can contribute to reproductive isolation and speciation [20].

**Figure 1:**
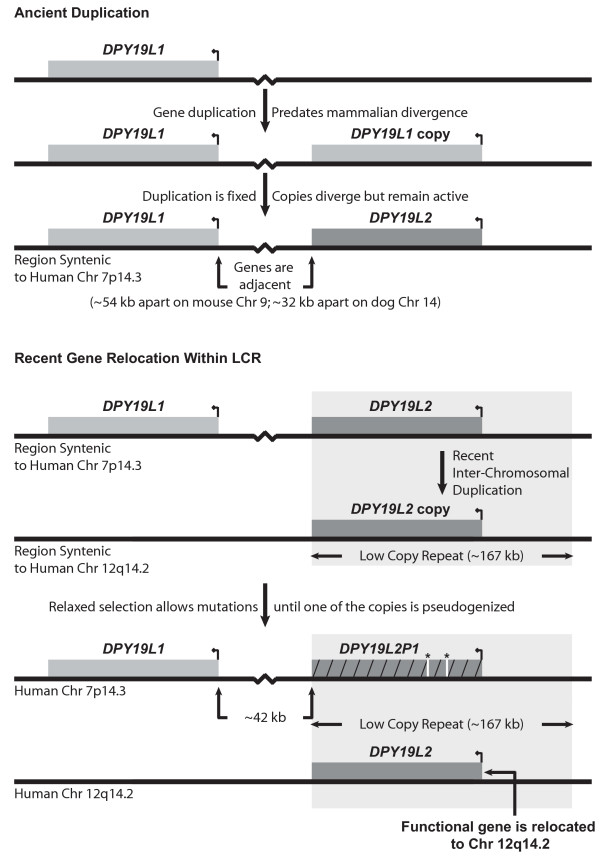
**Schematic illustration of the creation and relocation of the functional *DPY19L2 *gene**. Ancient duplication of *DPY19L1 *prior to mammalian divergence created a duplicate copy of the gene. The ancestral locus of *DPY19L2 *is defined by ascertaining the gene's location in non-primate mammals. Consistently, the gene is found adjacent to *DPY19L1 *in a region syntenic to human Chromosome 7, suggesting the ancient duplication was in tandem. Recent inter-chromosomal duplication (grey box surrounding *DPY19L2*) occurred creating a second copy of *DPY19L2 *within a region syntenic to human Chromosome 12. Sequence evolution introduced two STOP codons (asterisks above white bars) into the ancestral gene, leading to its pseudogenization (cross-hatching indicates pseudogene). Thus, functional *DPY19L2 *has been relocated from its ancestral location on Chromosome 7 to a descendant region on Chromosome 12 within a recent LCR.

Here we describe the relocation of the gene *DPY19L2 *within a set of LCRs (Fig. 1). Although this type of genome modification has been hypothesized [20], very few examples of this type of relocation have been documented [21,22]. We demonstrate the relocation of a functional gene through its duplication and subsequent pseudogenization within a LCR. Additionally, we characterize another functional gene, *DPY19L1*, and six pseudogenes within these LCRs. Both of these functional genes belong to a novel transmembrane gene family, *DPY19L*, containing four human genes, which has undergone both ancient and recent duplications. This paper investigates the evolution of these novel transcripts and describes the LCRs that are associated with the recent gene duplications and relocation.

## Results

### Physical organization of the low copy repeats

**Figure 2:**
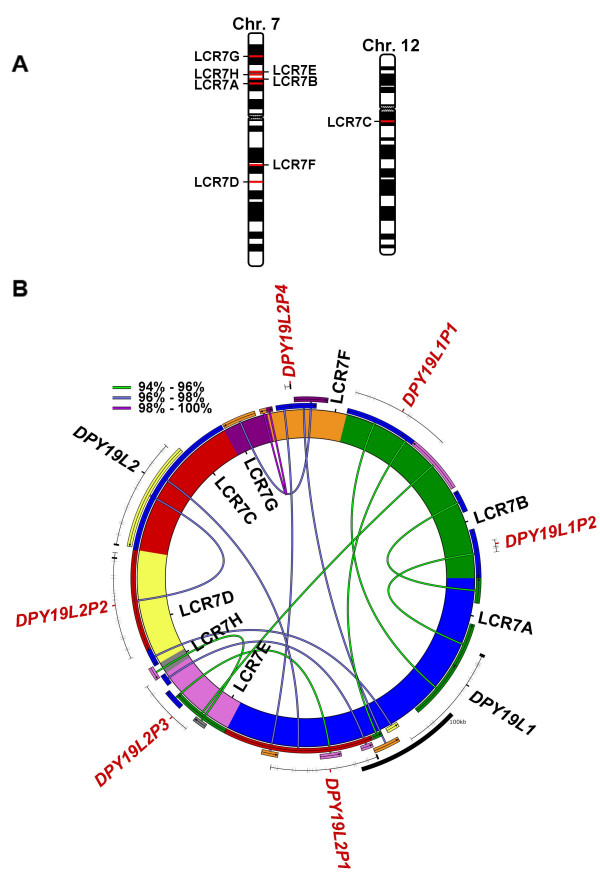
**Structure and organization of a set of LCRs on human Chromosomes 7 and 12**. A) An overview of the LCR regions showing their locations on ideograms of the two chromosomes. B) Structures of the eight LCR7 regions. The LCRs are depicted by the thick circle, where the size of the LCR is proportional to the size of the region in the circle. Duplicated segments, >5 kb with sequence identity >90%, are shown connected to the homologous region with the highest sequence identity. This best match approach was used to minimize redundant duplications and decrease the structural complexity. The colour of the lines connecting the two homologous sequences corresponds to the sequence identity between the two segments. Genes (black) and pseudogenes (red) are shown on an outer level of the circular figure. The black bar at the bottom right of the figure shows the scale of the circular segments, representing 100 kb of sequence.

A large duplication of sequence between 7p14.3 and 7p15.1 was identified during the annotation of human Chromosome 7 [23]. Blastn [24] searches against the **nr **database using these two regions as query sequences identified six additional loci containing large stretches of duplicated DNA. In total, eight regions were identified with >90% identity over >5 kb and were designated LCR7A through LCR7H based on their sizes. Seven of these regions are located on Chromosome 7, five on the p arm and two on the q arm, while one region is located on the q arm of Chromosome 12 (Fig. 2A; see Additional file 1).

To define the duplication boundaries and determine their structures, two programs, Blast 2 sequences [25] and mVISTA [26-28] were utilized. For these analyses, the sequences of the LCRs on Chromosome 7 were obtained from the Chromosome 7 annotation project sequence (CRA_TCAGchr7.v2) while the sequence on Chromosome 12 was acquired from NCBI's public Build 35 (see Additional file 1). Using the two programs we were able to determine the size and sequence content of the LCRs (see Additional file 2). We were also able to resolve the complex modular structure of the duplications, tracing much of the duplicated DNA back to LCR7A by a best match analysis (Fig. 2B). Overall, the eight LCRs comprise more than 1 Mb of duplicated DNA with sequence identities ranging from 94–98% (see Additional file 2).

### Identification of transcripts in low copy repeats

**Figure 3:**
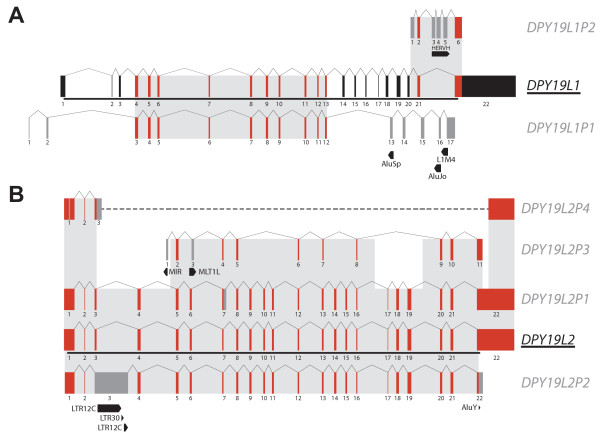
**Structures of the transcripts found within the set of LCRs**. Genes (labeled in black) and their pseudogenes (labeled in dark grey) are shown in relation to each other. Exons that are duplicated between transcripts are shown in red, while exons unique to a transcript are shown in black (genes) or dark grey (pseudogenes). Light grey boxes around exons represent regions of the transcripts, including introns, that have been duplicated. Open reading frames of the two functional genes are indicated by black bars under the transcripts. Repetitive elements contained within exons are shown in black below the transcripts. Intron sizes of all transcripts are based on the introns of the functional genes A) *DPY19L1 *and its two pseudogenes; B) *DPY19L2 *and its four pseudogenes. Dotted line in exon 3 of *DPY19L2P4 *represents continuity between the two parts of the exon.

We inspected the eight regions for known transcripts that had been duplicated during the expansion of the LCRs. We initially examined LCR7A, since it was the largest of the examined LCRs, and found two known transcripts (Fig. 3A, 3B). We confirmed their transcription and identified upstream exons by RT-PCR and 5' RACE on human testis RNA. All transcripts identified below were analyzed in a similar manner.

The first transcript from LCR7A [Genbank:AB020684] has 22 exons and a complete open reading frame (ORF) coding for 675 amino acids. We defined an ORF as complete when it codes for >100 amino acids and is uninterrupted between a start codon in the first exon and an inframe stop codon in the last exon. This is a conservative definition of a complete ORF and may exclude transcripts with functions that do not require the complete CDS, such as those undergoing neo- or subfunctionalization. Transcripts with complete ORFs were classified as genes. Although the function of AB020684 is unknown, *in silico *analysis indicated that it encodes a protein with homology to the *C. elegans *protein *DPY-19 *(see Additional files 3 and 4). Therefore, we called this gene *DPY19L1 *(Fig. 3A). Ten exons from this gene (exons 4 to 13) appear to be duplicated in a known transcript within LCR7B [Genbank:AK026768]. Along with these ten exons, AK026768 has an additional two upstream and five downstream exons that fall outside the duplicated sequence, giving it a total of 17 exons. However, unlike *DPY19L1*, this transcript does not appear to encode a complete ORF because it has STOP codons in exons 2 and 11. This transcript, which we named *DPY19L1P1 *(Fig. 3A), was classified as a pseudogene. We defined a pseudogene as a duplicated transcript that has lost its original function due to incomplete duplication or at least one interruption of the ORF.

The second transcript from LCR7A [Genbank:BC066987] also has 22 exons. This transcript does not have a complete ORF because it has STOP codons in exons 6 and 7. Duplications of all 22 exons of this transcript were seen in two other regions: LCR7C and LCR7D. The transcript in LCR7C [Genbank:AL833344] is a gene because it has a complete ORF coding for 758 amino acids.* In silico *analysis of this ORF again showed homology to the *C. elegan*s gene *DPY-19*, although with a lower percent identity (38% amino acid identity, compared with 43% identity for DPY19L1) (see Additional files 3 and 4). Therefore, we named this gene *DPY19L2 *(Fig. 3B). Like the second transcript on LCR7A, the transcript on LCR7D [Genbank:AL834175] has inframe STOP codons, caused by insertions of LTR repeats in exon 3. These inactivated transcripts on LCR7A and LCR7D are classified as pseudogenes, and we named them *DPY19L2P1 *and *DPY19L2P2 *respectively (Fig. 3B).

The two genes and three pseudogenes make up a set of duplicated known transcripts within the eight LCRs. To identify novel transcripts that belong within this set, we used the two coding genes as queries for UCSC BLAT searches [29] against the human genome Build 35 sequence. Using sequence identity to uncharacterized mRNA and EST transcripts, we were able to identify three additional pseudogenes that arose from partial duplications. One transcript in LCR7B, *DPY19L1P2*, shows high identity with *DPY19L1*, while two other transcripts, *DPY19L2P3 *in LCR7E and *DPY19L2P4 *in LCR7F, share high identity with *DPY19L2 *(Fig. 3A, 3B). The relationships between these transcripts are illustrated by an analysis of their phylogeny (see Additional file 5).

### Genomic relocation of DPY19L2

Although both *DPY19L1 *and *DPY19L2 *share homology to the *C. elegan*s gene *DPY-19 *and are within a set of related LCRs, these two genes are not recent duplicates of each other. We reached this conclusion because the region of LCR7A that contains *DPY19L1 *does not show high sequence identity with the region of LCR7C that contains *DPY19L2 *(Fig. 2B). Instead, we attribute their similarity to an ancient duplication that occurred prior to mammalian divergence. This is supported by two observations: the percent nucleotide identity between the two transcripts is ~76% (less than the 90% cutoff typically used for recent duplications [1,2]; see Additional file 3) and both genes are also present in mouse. The two mouse genes, *Dpy19l1 *and *Dpy19l2*, share ~72% nucleotide sequence identity with each other and are ~54.3 kb apart on mouse Chromosome 9 in a region syntenic to LCR7A on human Chromosome 7p14.3 (Fig. 1). Importantly, Blastn analysis indicates that these two genes, and their genomic regions, are unique in the mouse genome and do not appear to have been recently duplicated within LCRs. Notably, the functional *Dpy19l2 *is syntenic to the human pseudogene *DPY19L2P1*, not to the functional *DPY19L2 *on Chromosome 12. This indicates that the ancestral copy of *DPY19L2 *was present on Chromosome 7 (Fig. 1). This is confirmed by our analysis of the genes in dog and rat. Segmental duplication of this region onto Chromosome 12q14.2 established two copies of the functional gene. Initial functional redundancy relaxed the selection on one or both of the copies of this gene, allowing the fixation of an inactivating pseudogenization event. In this case, the ancestral copy on 7p14.3 was pseudogenized while the copy on 12q14.2 remained functional. Thus, *DPY19L2 *has undergone gene relocation within a recent LCR (Fig. 1).

### Neutral evolution of recently duplicated transcripts

We analyzed the three known pseudogenes within the LCRs, each of which contains a substantial duplication of the original ORF. We hypothesized that if these transcripts are no longer functional, their sequences would not be under selectional constraint. Hence, they would be evolving neutrally. We looked for evidence of selection by two methods, codon position bias of nucleotide changes and K_a_/K_s_; each assumes nucleotide changes accumulate evenly in the three codon positions during neutral evolution. As it is possible that truncation following duplication can result in a new gene, with purifying selection 5' and neutral evolution 3' of the new STOP codon, we selected the largest ORF in each of the pseudogenes for this analysis. In each case, the largest ORF observed corresponded to a truncation of the original ORF, rather than a frameshift.

Our first analysis looks at the distributions of nucleotide changes between the functional genes and the inactive transcripts and then compares them to the distributions of changes between the functional genes in human and mouse. In this analysis, the nucleotide changes in the largest ORF of the inactive transcripts are more equally distributed than the changes between the human and mouse functional genes (Table 1). As expected, the changes between the two functional genes are in a 3^rd^>1^st^>2^nd ^codon position distribution, which is indicative of purifying selection.

Distributions of nucleotide changes affect the synonymous (K_s_) and non-synonymous (K_a_) substitution rates. In purifying selection, we expect the synonymous rate to be much higher than the non-synonymous rates (K_a_/K_s_<1), whereas in neutral selection the rates are expected to be very similar. Much like the first analysis, we see that the substitution rate between the functional human and mouse genes suggests purifying selection, while the K_a_/K_s _values within the inactive genes' ORFs are much higher (Table 1). Most likely, this increase in K_a_/K_s _indicates relaxed or neutral selection of the pseudogenes.

### DPY19-like gene family

Along with *DPY19L1 *and *DPY19L2*, homology searches identified two other human genes that have amino acid identity to the *C. elegans *gene *DPY-19 *(see Additional files 3 and 4). These two genes, *DPY19L3 *and *DPY19L4*, are located on Chromosomes 19q13.11 and 8q22.1, respectively, and do not reside in any segmental duplications. Both of these transcripts are known genes spanning 18 exons and are covered by full-length mRNA transcripts ([Genbank:AK126757], encoding 716 amino acids, and [Genbank:AK123682], encoding 723 amino acids, respectively). Similar to *DPY19L1 *and *DPY19L2*, the functions of these genes are uncharacterized.

**Figure 4:**
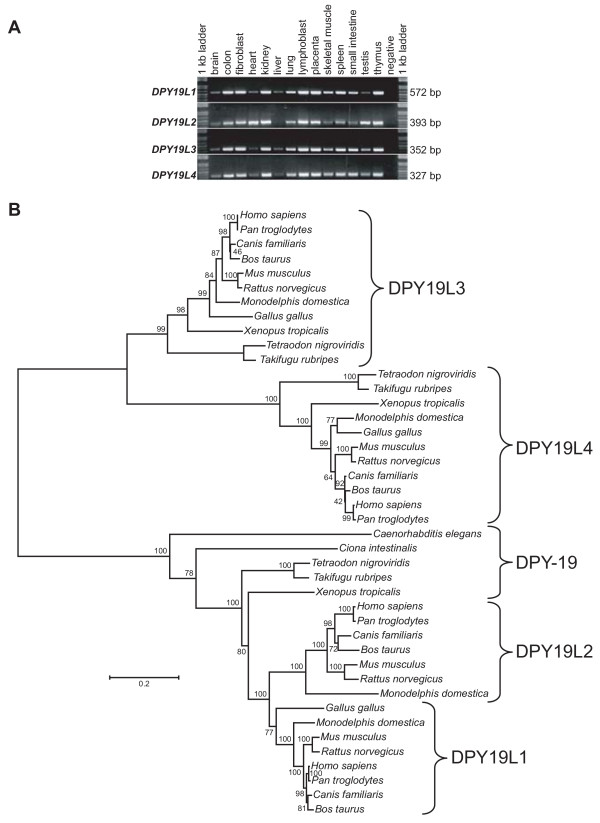
**Comparison of *DPY19L *family members**. A) RT-PCR analysis of the four human genes. B) Phylogenetic analysis of *DPY19L *gene family evolution in multiple animal species. An alignment of 42 protein sequences was created in ClustalW and input into MEGA3. The neighbour-joining tree was created using the Jones-Taylor-Thornton matrix for amino acid evolution and bootstrap values, calculated from 250 replications, are shown at internal nodes. Proteins are labeled by species and grouped into families (defined by brackets with family name on the right).

RT-PCR analysis of these homologous genes shows similar ubiquitous expression in all tissues examined (Fig. 4A). However, although the pattern of expression is highly similar between *DPY19L1*, *DPY19L3*, and *DPY19L4*, there appears to be some slight variations in the expression of *DPY19L2*. The majority of these differences can be attributed to the concomitant amplification of two pseudogenes, *DPY19L2P1 *and *DPY19L2P2*. These two pseudogenes share such high identity with the functional gene that we were unable to specifically amplify *DPY19L2*. By sequencing the bands from the RT-PCR experiment, we were able to determine the expression of *DPY19L2 *and the two pseudogenes by analyzing transcript specific paralogous sequence variants. *DPY19L2 *appears to be expressed to some degree in all tissues, while *DPY19L2P1 *is expressed in the brain, heart, placenta and testis and *DPY19L2P2 *is expressed in the fibroblast, lung, lymphoblast, spleen and testis. Superfluous pseudogene amplification can account for the increase in expression seen in certain tissues. However, other tissues, such as liver, skeletal muscle, spleen and small intestine, are shown to have significantly lower expression in *DPY19L2 *(Fig. 4A). This might indicate some differences in *DPY19L2 *gene regulation.

We used NCBI's BLAST [24] and UCSC's BLAT [29] tools to identify proteins homologous to *DPY-19 *in other animals (see Methods). These protein sequences were then aligned using ClustalW [30]. With the multiple alignment file, a neighbour-joining phylogenetic tree was constructed using MEGA3 [31] (Fig. 4B). As a test of inferred phylogeny, bootstrap values were calculated using 250 replications. From this analysis we can see that invertebrate animals such as nematodes (*C. elegans*) and deuterostomes (*C. intestinalis*) have a single *DPY-19 *gene, while all vertebrates examined have at least three homologous copies. This suggests that multiple duplications have occurred during vertebrate evolution. The first duplications appear to have occurred prior to the divergence of the fish lineage, giving rise to two additional copies, *DPY19L3 *and *DPY19L4*, which are present in all vertebrates. An additional duplication, generating *DPY19L2*, appears to have arisen prior to mammalian divergence. This gene appears in all mammals examined, including the metatherian opossum (*M. domestica*). However, it is unclear whether this duplication occurred before or after the divergence of chicken. Although chicken is lacking *DPY19L2*, one of its proteins is placed in the DPY19L1 clade (with a relatively high bootstrap value of 77%) rather than grouping with the DPY-19 proteins. This could indicate that the duplication leading to *DPY19L2 *has occurred before chicken divergence with subsequent gene loss in chicken (or the second copy may not be represented in the genome sequences available for the chicken due to the incomplete nature of its genome assembly). Alternatively, the duplication could have occurred after chicken divergence, with the chicken protein maintaining more similarity to DPY19L1 due to an accelerated evolution of *DPY19L2 *relative to *DPY19L1 *(longer branch lengths of DPY19L2 proteins are seen in Fig. 4B). Through these observations, we estimate that the duplication that generated *DPY19L2 *arose between 173 (*Marsupilia*) and 360 (*Lissamphibia*) MYA (based on divergence times in [32]). Additional trees were built using different phylogenetic methods (including minimum evolution and maximum parsimony) to confirm the observed phylogeny. Overall, these trees had a highly similar topology to the tree presented in Fig. 4B (data not shown).

### Structure and conservation of DPY-19 homologs

DPY-19 is a novel transmembrane protein that is required for the proper polarization and migration of neuroblasts in *C. elegans*. It has been shown to express weakly in the QL and QR neuroblasts, their neighbouring epidermal cells, and in dorsal and ventral body muscle cells [33]. To ensure that the four human homologues have similar structures to this gene, we compared their domain architectures using SMART [34] and Pfam [35]. DPY-19 does not have any predicted SMART or Pfam domains, but does have 10 predicted transmembrane domains, consistent with its characterization as a transmembrane protein. Similarly, the four human homologues have 9 to 11 predicted transmembrane domains, which suggest that they share a similar protein structure with DPY-19.

Using the multiple species amino acid alignment, we were able to identify putative motifs with high conservation using the program MEME [36]. Three putative motifs, each at least 50 amino acids in width and present in at least 40 of the 42 proteins, were identified by this program. All three putative domains are significantly more conserved at the amino acid level than the rest of the proteins (P-value < 0.0001; See Methods). However, two of these motifs contained one or more transmembrane domains in a subset of the proteins. Therefore, the conservation in these two regions is most likely due to the presence of a transmembrane domain. The third motif, which was the largest and most significant motif identified by MEME, does not match any known domains. This motif is present near the C-termini of the proteins and may represent a novel functionally relevant sequence (see Additional file 6).

## Discussion

Low copy repeats in extant genomes represent the most recent duplications of large stretches of DNA. In humans, sequence and cytogenetic data indicate that the commonly used thresholds are detecting LCRs that have arisen within the past 35 MY of primate evolution [1,2,37]. These active, and not uncommon, restructurings within genomes have important implications in molecular evolution. Genes duplicated within LCRs can diverge through substitutions or exon shuffling [38,39], allowing, in rare circumstances, novel functions to develop. Also, because of their high nucleotide sequence identity, copies of LCRs can act as substrates for large rearrangements, such as deletions, inversions and duplications (reviewed in [40]). Such large rearrangements are often found to cause genomic disorders, while less severe changes are theorized to be significant factors in genome evolution and speciation.

In this paper, we demonstrate that LCRs can also have a more subtle effect on genome evolution. We first characterize a set of LCRs on human Chromosomes 7 and 12. These LCRs have a complex modular structure with sequence identities ranging from 94–98% (Figure 2B; see Additional file 2). The exact order of the LCR expansion is difficult to ascertain due to this complexity, as well as the similarity in sequence identity between the regions. Even the branching order of the transcripts contained within the LCRs is not easily resolved due to their high sequence similarity (see Additional file 5). After the characterization of these regions and their transcripts, we then describe a novel example of functional gene relocation through duplication and subsequent pseudogenization within a LCR (Fig. 1). This is a type of small chromosomal rearrangement that has been implicated in evolution, and has been suggested to be involved in speciation events through population isolation followed by divergent resolution of duplicate genes [20,41]. Gene silencing has also been shown to relocate functional genes following whole genome duplication [21] or subtelomeric duplication [22] events. The example we report is the first characterization of functional gene relocation within a euchromatic LCR. We propose that, like rearrangements of DNA segments and gene structures, rearrangements in gene order during LCR creation and expansion are a noteworthy mechanism for genome evolution and divergence.

Unlike whole genome duplication, gene relocation following duplication within a LCR can modify a gene's genomic context. Specifically, *cis*-acting elements neighbouring the ancestral locus may be different from the *cis*-acting elements neighbouring the descendent locus, causing the positional effects on the gene to be altered. Thus, changes in expression and function are considered to be another consequence of gene relocation. Interestingly, the slight variation in *DPY19L2 *expression (Fig. 4A) could indicate a change in regulation. One interpretation of this finding is that the change in expression is caused by a difference in *cis*-acting elements between the functional gene's ancestral and descendant locations.

Our data is consistent with the concept that there are two major fates of gene duplication: retention of both copies or pseudogenization of one of the transcripts. Within the set of LCRs, we were able to identify two homologous functional genes, apparently created by a duplication event of a common ancestor homologous to the *C. elegans DPY-19 *gene. These genes illustrate how both copies of a gene duplicate can be retained. Preservation of both copies may be due to some selective advantage, either through increased dosage, neofunctionalization, or complementary degeneration leading to subfunctionalization.

These functional genes have been duplicated within LCRs, creating new transcripts. However, for both genes only a single transcript has remained active. The others, although transcribed, are all pseudogenes due to loss of coding potential through partial duplication or accumulation of deleterious mutations (Fig. 3A, 3B). This supports the notion that pseudogenization is the most common fate of gene duplication [8]. In addition, we tested for selection acting on the three duplicated pseudogenes containing a substantial ORF. In two of the three transcripts, selection was relaxed such that they appeared to be undergoing neutral selection. In the third transcript, the selection was relaxed relative to the functional gene, but some purifying selection, indicated by K_a_/K_s _<1, was still apparent (Table 1). It is important to note that an increased ratio may also represent a mixture of positive and negative selection. However, in combination these analyses show that the inactive transcripts appear to be undergoing neutral evolution, indicating that they have indeed lost their coding potential and are not being selectively maintained. This fits with the perception that most pseudogenes, like non-coding regions, are free of selection [42,43]. Evidence of selection in one transcript could indicate that it has some actively maintained function. However, the significantly relaxed selection in this case may suggest a recent inactivation that has yet to accumulate enough mutations for neutral selection to be distinguished. In any case, we expect the non-functional remnants of gene duplication to continue to diverge from the original copy, along with the other non-coding regions of the LCR, until eventually all evidence of duplication is erased.

The two functional genes, along with two additional homologues outside of the LCRs, constitute a novel gene family that we designate as the *DPY19L *family. Although the function of this family is unknown, each member has a large complement of predicted transmembrane domains, indicating that they are functional within one of the cellular membranes. Along with these domains, this family contains a novel putative motif of high conservation (see Additional file 6). The high sequence conservation in this putative motif suggests that its sequence may represent a novel functionally important domain that has been maintained throughout the evolution of this gene family. Interestingly, *DPY19L *family members have undergone multiple duplications throughout their evolution. Ancient duplications before the divergence of fish led to the creation of two paralogues (*DPY19L3 *and *DPY19L4*), while an additional duplication after the divergence of amphibia, yet before the divergence of mammals, created another gene (*DPY19L2*) (Fig. 4B). Analyses of the human genome sequence indicate that the duplication of these genes is an ongoing process. In fact, all four human paralogues show evidence for relatively recent gene duplication. *DPY19L1 *and *DPY19L2 *are both contained in recent low copy repeats, while *DPY19L3 *and *DPY19L4 *appear to have processed pseudogenes with duplications of at least 3 exons. Duplications of genes are known to play important roles in gene evolution. The recent duplications of all four human genes suggest that the *DPY19L *gene family is still evolving and may be an important family in the divergence of species.

## Conclusion

The catalogue of LCRs provides an excellent resource for the study of genomic evolution. By characterizing the relationship between sets of LCRs and the genes they contain, we can attempt to understand the mechanisms behind these rearrangements and how they affect evolution. LCRs appear to influence genome composition by affecting genomic sequence structure, gene content and gene order. The last of these influences has not been vigorously investigated. Although this is one of the first observations showing a change in gene location with a set of LCRs, we expect that many other examples are present within LCR-rich genomes, and their identification could lead to a better understanding of genome divergence and speciation.

## Methods

### *In silico *identification of related LCRs

We queried masked genomic sequence against NCBI's nonredundant (**nr**) and high-throughput genomic sequence (**htgs**) databases using Blastn [24] to identify related copies of the LCRs. Eight regions >5 kb with ≥90% sequence identity were detected. Complete sequences for each of the LCRs was then obtained; for LCRs on Chromosome 7 we used sequence from the Chromosome 7 annotation project (CRA_TCAGchr7.v2), while the LCR sequence on Chromosome 12 was acquired from NCBI's public Build 35. Duplication boundaries were then determined by comparing the regions using Blast 2 sequence [25] and mVISTA [26-28]. Using the Blast 2 sequence results, we used a C++ program (CircleGraph, H. Wagenaar, unpubl.) to visually display the relationships between each of the LCRs (Fig. 2b). For this analysis, modules, or discrete regions of high identity, were concatenated when they were <5 kb apart or separated by repetitive elements, as long as the modules maintained their orientations between copies. Also, modules <5 kb were eliminated from the analysis, causing some boundaries (LCR7F in Fig. 2B) to appear uncovered by duplications.

### Transcript characterization

Transcripts within the LCRs were identified using full-length mRNAs and spliced ESTs annotated by UCSC's Genome Browser [29]. RT-PCR was used to confirm the expression of transcripts annotated as 'Known Genes'. For novel transcripts, uncharacterized mRNA and ESTs, along with exons predicted by homology analyses, were amplified by RT-PCR and sequenced to determine the complete sequence. Rapid amplification of cDNA ends (RACE) was employed in cases where the 5' and/or 3' gene sequence was uncharacterized. RACE experiments were conducted using marathon-ready cDNA (Clontech) according to manufacturer's instructions. Primer sequences used for these experiments are available upon request.

Genomic organization of each transcript was determined by probing the sequence against the human genome using UCSC's BLAT tool (Fig. 3). Open reading frames (ORF) and protein sequences were determined through conceptual 6-frame translation using NCBI ORF finder [44]. Transcripts having an ORF coding for >100 amino acids, and spanning the entire transcript, from the first to last exon, were annotated as genes. Transcripts not meeting these criteria were classified as pseudogenes.

DNA sequences of the pseudogenes were manually aligned against the ORFs of the two functional genes. These transcripts were truncated such that exons that showed no homology to the functional genes were removed from the alignment. *DPY19L3 *was also aligned with these sequences and was used as an outgroup in the phylogenetic analysis. This alignment was converted to nexus format and analyzed using PAUP software [45]. A maximum likelihood tree was constructed using the general time reversible plus gamma (GTR+G) model of evolution and bootstrapped with 100 replicates (parameters for nucleotide composition, substitution rates and gamma shape were estimated by PAUP) (see Additional file 5).

Expression analyses of the four human *DPY19L *genes were performed using RT-PCR in 14 tissues. Primer sequences and conditions are available upon request. For *DPY19L2*, each of the products was purified using microCLEAN (Microzone) and sequenced to determine contamination of the *DPY19L2 *expression with pseudogene amplification. Pseudogene amplification was qualified by assessing the presence or absence of transcript specific paralagous sequence variants.

### Homology and evolutionary analyses

Using the BLAST [24] and BLAT [29] tools, we were able to identify human paralogues of the two protein coding genes detected in our set of LCRs. In addition, we used these same programs to search for orthologues of each of the human genes in a wide range of organisms. Orthologues were identified using BLAT against genome assemblies on the UCSC Genome Bioinformatics webpage [46]. Where no known orthologue is annotated, we assembled transcripts using mRNAs, ESTs and gene predictions. When no transcript information was present, genomic sequences were used to identify conserved exons, taking into account intron/exon boundaries. The trace archive database (NCBI) was searched when gaps in genome assemblies were encountered. ClustalW [30] was used to align the assembled protein homologues and MEGA3 [31] was used to construct a neighbour-joining phylogenetic tree. The neighbour-joining tree was created using the Jones-Taylor-Thornton matrix [47] for amino acid evolution and bootstrap values were calculated with 250 replications. Additional trees were built, using alternate phylogenetic methods (neighbour-joining trees under various models of evolution, minimum evolution trees and maximum parsimony trees) to verify the observed phylogeny.

We also used MEGA3 to determine the evolution of the recently duplicated transcripts. We first aligned the coding nucleotides of a gene against the longest homologous ORF in its pseudogene(s) using ClustalW. For this analysis we only used 'Known' transcripts. In the novel transcripts, the ORFs were too short to show significant results. To determine the codon position bias we used MEGA3 to calculate the nucleotide p-distance between the transcripts at each of the coding positions. Next, we used the Nei-Gojobori [48] p-distance method to calculate the frequency of synonymous substitutions (K_s_), the frequency of non-synonymous substitutions (K_a_) and the ratio between them (K_a_/K_s_).

### Protein structure analyses

Protein domain architectures, including transmembrane domains, were determined using SMART [34] and Pfam [35]. Putative functionally relevant motifs were identified using the program MEME [36]. Statistical analysis was performed on these sequences to determine if the predicted motifs were more highly conserved between species. We used MEGA3 to calculate amino acid and nucleotide p-distances inside and outside these motifs, while testing for significant differences with a Wilcoxon signed rank test.

## Authors' contributions

ARC participated in the design of the study, performed the molecular analyses and drafted the manuscript. JC participated in the design and coordination of the study. SWS conceived of the study, participated in its design and coordination and helped to draft the manuscript. All authors read and approved the final manuscript.

## Supplementary Material

Additional File 1 **Supplementary Table 1: Coordinates of low copy repeats**. This file lists the coordinates, in both NCBI Build 35 (hg17) and CRA_TCAGchr7.v2, of each LCR described in these analyses. Underlined coordinates indicate sequences used in the analyses, with the size of the region in brackets. Click here for file

Additional File 2 **Supplementary Table 2: Pairwise identity comparisons between the eight LCR regions**. This table lists the modified percent match value calculated between each identified LCR in this analysis. Click here for file

Additional File 3 **Supplementary Table 3: Nucleotide identity comparisons**. This file shows the nucleotide identities calculated from mVISTA (using SLAGAN alignment program) for the human genes (*DPY19L1 *through *DPY19L4*) and the *C. elegans *gene (*DPY-19*). Click here for file

Additional File 4 **Supplementary Table 4: Amino acid identity comparisons**. This file shows the amino acid identities and positives from Blast 2 Sequences for the human proteins (DPY19L1 through DPY19L4) and the *C. elegans *protein (DPY-19). Click here for file

Additional File 5 **Supplementary Figure 1: DNA-based phylogeny of the gene and pseudogene transcripts**. This file shows a maximum likelihood tree that was created in PAUP using a general time reversible plus gamma (α = 1.5090) model of sequence evolution. *DPY19L3 *was used as an outgroup to root the tree. Bootstrap values were calculated using 100 replicates and are shown at the nodes. Click here for file

Additional File 6 **Supplementary Figure 2: Putative novel domain identified by MEME**. This file shows one of three motifs identified by MEME. This is the only motif that does not contain a transmembrane domain. Amino acids conserved in >90% of the proteins (at least 38 of 42 sequences) are shaded (grey when similarity groups are used). Asterisks (*) denote the 23 amino acids that have >90% identity without using similarity groups. Click here for file

## Figures and Tables

**Table 1:** Nucleotide change analysis showing neutral evolution of pseudogenes

			Nucleotide p-distance at Codon Position			
				
Functional gene	vs	Pseudogene or Mouse gene	1^st^	2^nd^	3^rd^	K_a_/K_s_
*DPY19L1*		*DPY19L1P1*	0.027	0.014	0.038	0.4
*DPY19L1*		*Dpy19l1*	0.038	0.035	0.375	0.1
*DPY19L2*		*DPY19L2P1*	0.035	0.026	0.025	1.0
*DPY19L2*		*Dpy19l2*	0.158	0.101	0.347	0.3
*DPY19L2*		*DPY19L2P2*	0.065	0.034	0.029	1.3
*DPY19L2*		*Dpy19l2*	0.155	0.108	0.346	0.3
