# Supplementary material for: Duplication and relocation of the functional DPY19L2 gene within low copy repeats
Source: BMC Genomics. 2006 Mar 9;7:45. doi: 10.1186/1471-2164-7-45 (PMC1475853; doi:10.1186/1471-2164-7-45)
Supplement: Additional File 1 — Supplementary Table 1: Coordinates of low copy repeats. This file lists the coordinates, in both NCBI Build 35 (hg17) and CRA_TCAGchr7.v2, of each LCR described in these analyses. Underlined coordinates indicate sequences used in the analyses, with the size of the region in brackets. [file 1471-2164-7-45-S1.doc]

**Supplementary Table 1:** Coordinates of low copy repeats. Underlined coordinates indicate sequences used in the analyses with the size of the region in brackets below.

|  | **NCBI Build 35 (hg17) coordinates** | **CRA_TCAGchr7v2 coordinates** |
| --- | --- | --- |
| LCR7A | chr7:34666103-35054370 | chr7:34937870-35320224  (382355 bp) |
| **LCR7B** | chr7:32430046-32677114 | chr7:32701327-32948399  (247073 bp) |
| **LCR7C** | chr12:62237938-62405407  (167470 bp) | N/A |
| **LCR7D** | chr7:102391745-102520392 | chr7:102158160-102286807  (128648 bp) |
| **LCR7E** | chr7:29463807-29555602 | chr7:29740752-29832271  (91520 bp) |
| **LCR7F** | chr7:89370640-89459212 | chr7:89056761-89145330  (88570 bp) |
| **LCR7G** | chr7:22302820-22353930 | chr7:22580668-22631790  (51123 bp) |
| **LCR7H** | chr7:30097635-30110750 | chr7:30374161-30387273  (13113 bp) |
