# Supplementary material for: Duplication and relocation of the functional DPY19L2 gene within low copy repeats
Source: BMC Genomics. 2006 Mar 9;7:45. doi: 10.1186/1471-2164-7-45 (PMC1475853; doi:10.1186/1471-2164-7-45)
Supplement: Additional File 2 — Supplementary Table 2: Pairwise identity comparisons between the eight LCR regions. This table lists the modified percent match value calculated between each identified LCR in this analysis. [file 1471-2164-7-45-S2.doc]

**Supplementary Table 2:** Pairwise identity comparisons between the eight LCR regions. Calculated value is a modified version of the percent match computation:

modified percent match = matches/(matches+mismatches+gaps<5bp)

|  | **LCR7A** | **LCR7B** | **LCR7C** | **LCR7D** | **LCR7E** | **LCR7F** | **LCR7G** | **LCR7H** |
| --- | --- | --- | --- | --- | --- | --- | --- | --- |
| LCR7A |  |  |  |  |  |  |  |  |
| **LCR7B** | 94.92% |  |  |  |  |  |  |  |
| **LCR7C** | 97.20% | 94.12% |  |  |  |  |  |  |
| **LCR7D** | 97.04% | 94.03% | 96.99% |  |  |  |  |  |
| **LCR7E** | 96.07% | 97.19% | 94.80% | 94.85% |  |  |  |  |
| **LCR7F** | 97.13% | 94.48% | 97.04% | 97.47% | 94.82% |  |  |  |
| **LCR7G** | 97.02% | 94.84% | N/A | 97.21% | N/A | 97.79% |  |  |
| **LCR7H** | N/A | 95.04% | N/A | N/A | 95.12% | N/A | N/A |  |
