# Supplementary material for: Duplication and relocation of the functional DPY19L2 gene within low copy repeats
Source: BMC Genomics. 2006 Mar 9;7:45. doi: 10.1186/1471-2164-7-45 (PMC1475853; doi:10.1186/1471-2164-7-45)
Supplement: Additional File 3 — Supplementary Table 3: Nucleotide identity comparisons. This file shows the nucleotide identities calculated from mVISTA (using SLAGAN alignment program) for the human genes (DPY19L1 through DPY19L4) and the C. elegans gene (DPY-19). [file 1471-2164-7-45-S3.doc]

**Supplementary Table 3: Nucleotide identities from mVISTA (using SLAGAN alignment program) for the human genes (*DPY19L1* through *DPY19L4*) and the *C. elegans* gene (*DPY-19*).**

| Genes | *DPY-19* | *DPY19L1* | *DPY19L2* | *DPY19L3* | *DPY19L4* |
| --- | --- | --- | --- | --- | --- |
| *DPY-19* |  |  |  |  |  |
| *DPY19L1* | 53% |  |  |  |  |
| *DPY19L2* | 52% | 76% |  |  |  |
| *DPY19L3* | No alignment | No alignment | No alignment |  |  |
| *DPY19L4* | No alignment | No alignment | No alignment | 54% |  |
