# Supplementary material for: Duplication and relocation of the functional DPY19L2 gene within low copy repeats
Source: BMC Genomics. 2006 Mar 9;7:45. doi: 10.1186/1471-2164-7-45 (PMC1475853; doi:10.1186/1471-2164-7-45)
Supplement: Additional File 4 — Supplementary Table 4: Amino acid identity comparisons. This file shows the amino acid identities and positives from Blast 2 Sequences for the human proteins (DPY19L1 through DPY19L4) and the C. elegans protein (DPY-19). [file 1471-2164-7-45-S4.doc]

**Supplementary Table 4: Amino acid identities (top) and positives (bottom) from Blast 2 Sequences for the human proteins (DPY19L1 through DPY19L4) and the *C. elegans* protein (DPY-19).**

| Proteins | DPY-19 | DPY19L1 | DPY19L2 | DPY19L3 | DPY19L4 |
| --- | --- | --- | --- | --- | --- |
| DPY-19 |  |  |  |  |  |
| DPY19L1 | 43% (272/628)  62% (397/628) |  |  |  |  |
| DPY19L2 | 38% (257/660)  58% (393/660) | 68% (444/645)  82% (536/645) |  |  |  |
| DPY19L3 | 30% (200/656)  47% (316/656) | 30% (209/685)  47% (327/685) | 28% (200/693)  47% (336/693) |  |  |
| DPY19L4 | 24% (162/666)  43% (290/666) | 25% (165/638)  47% (308/638) | 25% (181/701)  45% (325/701) | 43% (311/718)  64% (468/718) |  |
