# Supplementary figures and images for: Duplication and relocation of the functional DPY19L2 gene within low copy repeats
Source: BMC Genomics. 2006 Mar 9;7:45. doi: 10.1186/1471-2164-7-45 (PMC1475853; doi:10.1186/1471-2164-7-45)

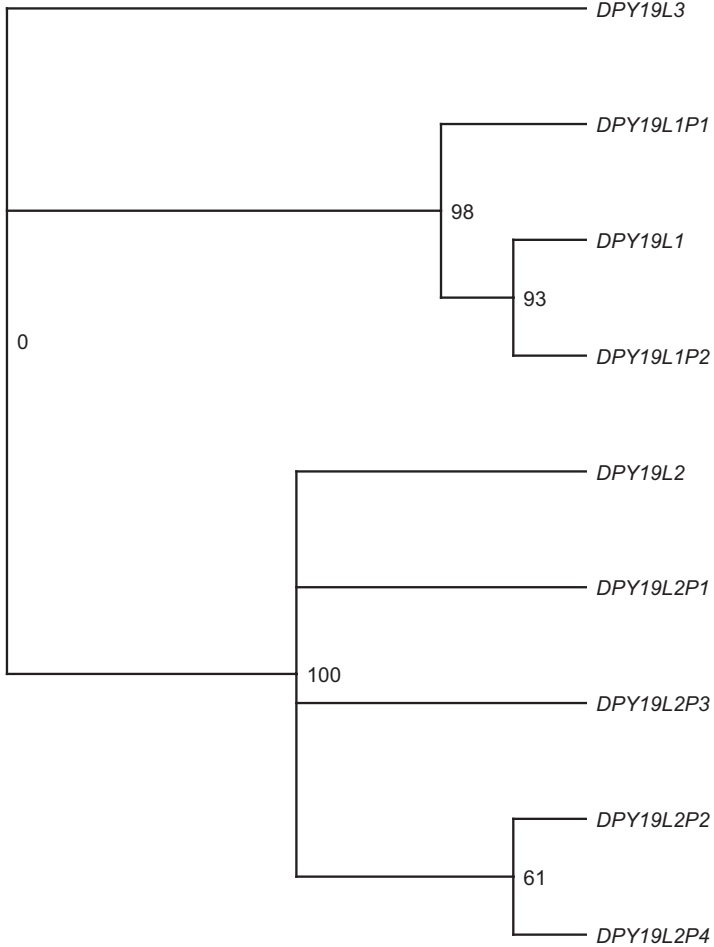

Supplement: Additional File 5 — Supplementary Figure 1: DNA-based phylogeny of the gene and pseudogene transcripts. This file shows a maximum likelihood tree that was created in PAUP using a general time reversible plus gamma (α = 1.5090) model of sequence evolution. DPY19L3 was used as an outgroup to root the tree. Bootstrap values were calculated using 100 replicates and are shown at the nodes. [file 1471-2164-7-45-S5.pdf]
