# Supplementary material for: Duplication and relocation of the functional DPY19L2 gene within low copy repeats
Source: BMC Genomics. 2006 Mar 9;7:45. doi: 10.1186/1471-2164-7-45 (PMC1475853; doi:10.1186/1471-2164-7-45)
Supplement: Additional File 6 — Supplementary Figure 2: Putative novel domain identified by MEME. This file shows one of three motifs identified by MEME. This is the only motif that does not contain a transmembrane domain. Amino acids conserved in >90% of the proteins (at least 38 of 42 sequences) are shaded (grey when similarity groups are used). Asterisks (*) denote the 23 amino acids that have >90% identity without using similarity groups. [file 1471-2164-7-45-S6.pdf]

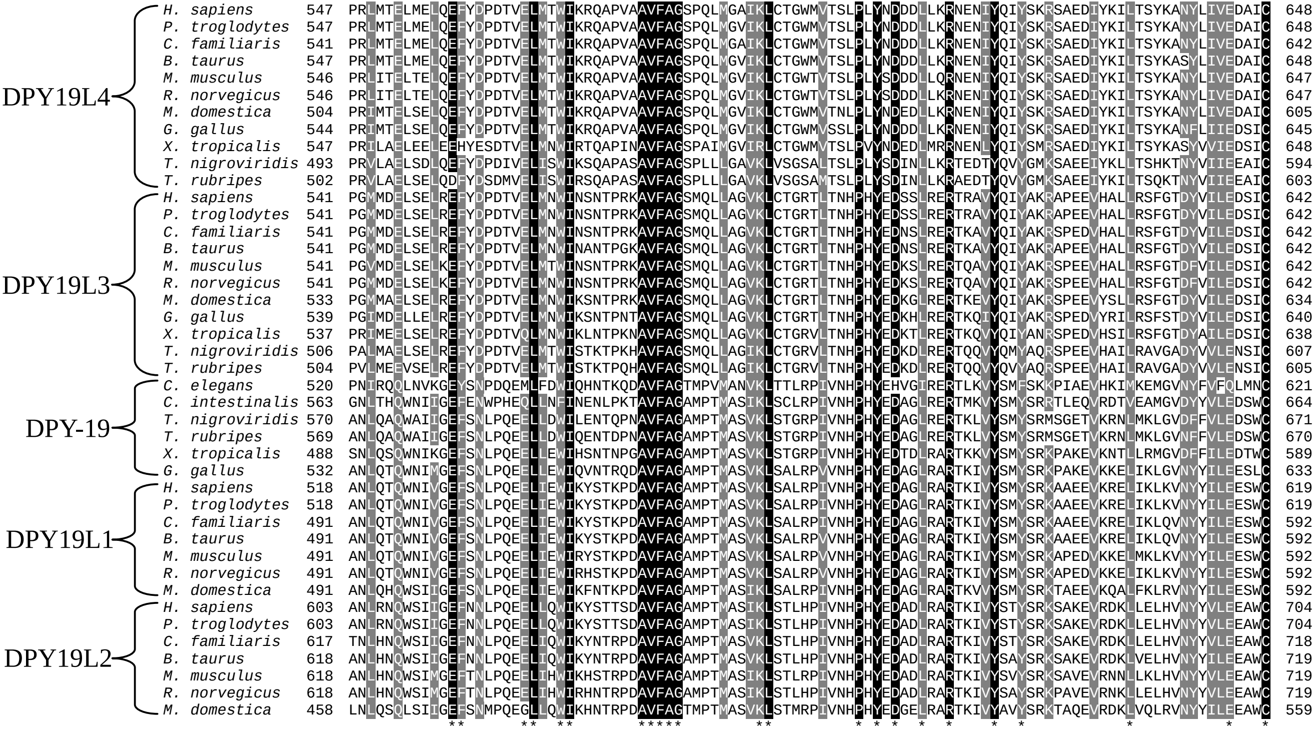
DPY19L4
H. sapiens 547 PRLMTELMELQEFYDPDTVELMTWIKRQAPVAAVFAGSPQLMGAIKLCTGWMVTSLPLYNDDDLLKRNENIYQIYSKRSAEDIYKILTSYKANYLIVEDAIC 648
P. troglodytes 547 PRLMTELMELQEFYDPDTVELMTWIKRQAPVAAVFAGSPQLMGVIKLCTGWMVTSLPLYNDDDLLKRNENIYQIYSKRSAEDIYKILTSYKANYLIVEDAIC 648
C. familiaris 541 PRLMTELMELQEFYDPDTVELMTWIKRQAPVAAVFAGSPQLMGAIKLCTGWMVTSLPLYNDDDLLKRNENIYQIYSKRSAEDIYKILTSYKANYLIVEDAIC 642
B. taurus 547 PRLMTELMELQEFYDPDTVELMTWIKRQAPVAAVFAGSPQLMGVIKLCTGWMVTSLPLYNDDDLLKRNENIYQIYSKRSAEDIYKILTSYKASYLIVEDAIC 648
M. musculus 546 PRLITELTELQEFYDPDTVELMTWIKRQAPVAAVFAGSPQLMGVIKLCTGWTVTSLPLYSDDDLLQRNENIYQIYSKRSAEDIYKILTSYKANYLIVEDAIC 647
R. norvegicus 546 PRLITELTELQEFYDPDTVELMTWIKRQAPVAAVFAGSPQLMGVIKLCTGWTVTSLPLYSDDDLLKRNENIYQIYSKRSAEDIYKILTSYKANYLIVEDAIC 647
M. domestica 504 PRIMTELSELQEFYDPDTVELMTWIKRQAPVAAVFAGSPQLMGVIKLCTGWMVTNLPLYNDEDLLKRNENIYQIYSKRSAEDIYKILTSYKANYLIVEDAIC 605
G. gallus 544 PRIMTELSELQEFYDPDTVELMTWIKRQAPVAAVFAGSPQLMGVIKLCTGWMVSSLPLYNDDDLLKRNENIYQIYSKRSAEDIYKILTSYKANFLIIEDSIC 645
X. tropicalis 547 PRILAELEELEEHYESDTVELMNWIRTQAPINAVFAGSPAIMGVIRLCTGWMVTSLPVYNDEDLMRRNENLYQIYSMRSAEDIYKILTSYKASYVVIEDSIC 648
T. nigroviridis 493 PRVLAELSDLQEFYDPDIVELISWIKSQAPASAVFAGSPLLLGAVKLVSGSALTSLPLYSDINLLKRTEDTYQVYGMKSAEEIYKLLTSHKTNYVIIEEAIC 594
T. rubripes 502 PRVLAELSELQDFYDSDMVELISWIRSQAPASAVFAGSPLLLGAVKLVSGSAMTSLPLYSDINLLKRAEDTYQVYGMKSAEEIYKILTSQKTNYVIIEEAIC 603
DPY19L3
H. sapiens 541 PGMMDELSELREFYDPDTVELMNWINSNTPRKAVFAGSMQLLAGVKLCTGRTLTNHPHYEDSSLRERTRAVYQIYAKRAPEEVHALLRSFGTDYVILEDSIC 642
P. troglodytes 541 PGMMDELSELREFYDPDTVELMNWINSNTPRKAVFAGSMQLLAGVKLCTGRTLTNHPHYEDSSLRERTRAVYQIYAKRAPEEVHALLRSFGTDYVILEDSIC 642
C. familiaris 541 PGMMDELSELREFYDPDTVELMNWINSNTPRKAVFAGSMQLLAGVKLCTGRTLTNHPHYEDNSLRERTKAVYQIYAKRSPEDVHALLRSFGTDYVILEDSIC 642
B. taurus 541 PGMMDELSELREFYDPDTVELMNWINANTPGKAVFAGSMQLLAGVKLCTGRTLTNHPHYEDNSLRERTKAVYQIYAKRAPEEVHALLRSFGTDYVILEDSIC 642
M. musculus 541 PGVMDELSELKEFYDPDTVELMTWINSNTPRKAVFAGSMQLLAGVKLCTGRTLTNHPHYEDKSLRERTQAVYQIYAKRSPEEVHALLRSFGTDFVILEDSIC 642
R. norvegicus 541 PGMMDELSELKEFYDPDTVELMNWINSNTPRKAVFAGSMQLLAGVKLCTGRTLTNHPHYEDKSLRERTQAVYQIYAKRSPEEVHALLRSFGTDFVILEDSIC 642
M. domestica 533 PGMMAELSELREFYDPDTVELMNWIKSNTPRKAVFAGSMQLLAGVKLCTGRTLTNHPHYEDKGLRERTKEVYQIYAKRSPEEVYSLLRSFGTDYVILEDSIC 634
G. gallus 539 PGIMDELLELREFYDPDTVELMNWIKSNTPNTAVFAGSMQLLAGVKLCTGRTLTNHPHYEDKHLRERTKQIYQIYAKRSPEDVYRILRSFSTDYVILEDSIC 640
X. tropicalis 537 PRIMEELSELREFYDPDTVQLMNWIKLNTPKNAVFAGSMQLLAGVKLCTGRVLTNHPHYEDKTLRERTKQVYQIYANRSPEDVHSILRSFGTDYAILEDSIC 638
T. nigroviridis 506 PALMAELSELREFYDPDTVELMTWISTKTPKHAVFAGSMQLLAGIKLCTGRVLTNHPHYEDKDLRERTQQVYQMYAQRSPEEVHAILRAVGADYVVLENSIC 607
T. rubripes 504 PVLMEEVSELREFYDPDTVELMTWISTKTPQHAVFAGSMQLLAGIKLCTGRVLTNHPHYEDKDLRERTQQVYQVYAQRSPEEVHAILRAVGADYVVLENSIC 605
DPY-19
C. elegans 520 PNIRQQLNVKGEYSNPDQEMLFDWIQHNTKQDAVFAGTMPVMANVKLTTLRPIVNHPHYEHVGIRERTLKVYSMFSKKPIAEVHKIMKEMGVNYFVFQLMNC 621
C. intestinalis 563 GNLTHQWNIIGEFENWPHEQLLNFINENLPKTAVFAGAMPTMASIKLSCLRPIVNHPHYEDAGLRERTMKVYSMYSRRTLEQVRDTVEAMGVDYVVLEDSWC 664
T. nigroviridis 570 ANLQAQWAIIGEFSNLPQEELLDWILENTQPNAVFAGAMPTMASVKLSTGRPIVNHPHYEDAGLRERTKLVYSMYSRMSGETVKRNLMKLGVDFFVLEDSWC 671
T. rubripes 569 ANLQAQWAIIGEFSNLPQEELLDWIQENTDPNAVFAGAMPTMASVKLSTGRPIVNHPHYEDAGLRERTKLVYSMYSRMSGETVKRNLMKLGVNFFVLEDSWC 670
X. tropicalis 488 SNLQSQWNIKGEFSNLPQEELLEWIHSNTNPGAVFAGAMPTMASVKLSTGRPIVNHPHYEDTDLRARTKKVYSMYSRKPAKEVKNTLLRMGVDFFILEDTWC 589
G. gallus 532 ANLQTQWNIMGEFSNLPQEELLEWIQVNTRQDAVFAGAMPTMASVKLSALRPVVNHPHYEDAGLRARTKIVYSMYSRKPAKEVKKELIKLGVNYYILEESLC 633
DPY19L1
H. sapiens 518 ANLQTQWNIVGEFSNLPQEELIEWIKYSTKPDAVFAGAMPTMASVKLSALRPIVNHPHYEDAGLRARTKIVYSMYSRKAAEEVKRELIKLKVNYYILEESWC 619
P. troglodytes 518 ANLQTQWNIVGEFSNLPQEELIEWIKYSTKPDAVFAGAMPTMASVKLSALRPIVNHPHYEDAGLRARTKIVYSMYSRKAAEEVKRELIKLKVNYYILEESWC 619
C. familiaris 491 ANLQTQWNIVGEFSNLPQEELIEWIKYSTKPDAVFAGAMPTMASVKLSALRPIVNHPHYEDAGLRARTKIVYSMYSRKAAEEVKRELIKLQVNYYILEESWC 592
B. taurus 491 ANLQTQWNIVGEFSNLPQEELIEWIKYSTKPDAVFAGAMPTMASVKLSALRPVVNHPHYEDAGLRARTKIVYSMYSRKAAEEVKRELIKLQVNYYILEESWC 592
M. musculus 491 ANLQTQWNIVGEFSNLPQEELIEWIRYSTKPDAVFAGAMPTMASVKLSALRPVVNHPHYEDAGLRARTKIVYSMYSRKAPEDVKKELMKLKVNYYILEESWC 592
R. norvegicus 491 ANLQTQWNIVGEFSNLPQEELIEWIRHSTKPDAVFAGAMPTMASVKLSALRPVVNHPHYEDAGLRARTKIVYSMYSRKAPEDVKKELIKLKVNYYILEESWC 592
M. domestica 491 ANLQHQWSIIGEFSNLPQEELIEWIKFNTKPDAVFAGAMPTMASIKLSALRPIVNHPHYEDAGLRARTKVVYSMYSRKTAEEVKQALFKLRVNYYILEESWC 592
DPY19L2
H. sapiens 603 ANLRNQWSIIGEFNNLPQEELLQWIKYSTTSDAVFAGAMPTMASIKLSTLHPIVNHPHYEDADLRARTKIVYSTYSRKSAKEVRDKLLELHVNYYVLEEAWC 704
P. troglodytes 603 ANLRNQWSIIGEFNNLPQEELLQWIKYSTTSDAVFAGAMPTMASIKLSTLHPIVNHPHYEDADLRARTKIVYSTYSRKSAKEVRDKLLELHVNYYVLEEAWC 704
C. familiaris 617 TNLHNQWSIIGEFNNLPQEELIQWIKYNTRPDAVFAGAMPTMASVKLSTLHPIVNHPHYEDADLRARTKIVYSTYSRKSAKEVRDKLLELHVNYYVLEEAWC 718
B. taurus 618 ANLHNQWSIIGEFNNLPQEELIQWIKYNTRPDAVFAGAMPTMASVKLSTLHPIVNHPHYEDADLRARTKIVYSAYSRKSAKEVRDKLVELHVNYYILEEAWC 719
M. musculus 618 ANLHNQWSIMGEFTNLPQEELIHWIKHSTRPDAVFAGAMPTMASIKLSTLRPIVNHPHYEDADLRARTKIVYSVYSRKSAVEVRNNLLKLHVNYYVLEEAWC 719
R. norvegicus 618 ANLHNQWSIMGEFTNLPQEELIHWIRHNTRPDAVFAGAMPTMASIKLSTLHPIVNHPHYEDADLRARTKIVYSAYSRKPAVEVRNKLLELHVNYYVLEEAWC 719
M. domestica 458 LNLQSQLSIIGEFSNMPQEGLLQWIKHNTRPDAVFAGTMPTMASVKLSTMRPIVNHPHYEDGELRARTKIVYAVYSRKTAQEVRDKLVQLRVNYYILEEAWC 559
** ** ** ***** ** * * * * * * * * * *
